# Splenic artery embolisation for blunt splenic trauma: 10 years of practice at a trauma centre

**DOI:** 10.1308/rcsann.2023.0035

**Published:** 2023-06-27

**Authors:** B Jones, AS Elbakri, C Murrills, P Patil, J Scollay

**Affiliations:** NHS Tayside, UK

**Keywords:** Spleen, Trauma, Splenectomy, Embolisation – Therapeutic

## Abstract

**Introduction:**

Splenic artery embolisation (SAE) has transformed the management of splenic trauma. The aim of this study was to review the outcomes and postprocedural management of blunt splenic trauma patients treated with SAE at a trauma centre over a 10-year period.

**Methods:**

Details of patients undergoing SAE for blunt trauma between January 2012 and January 2022 were acquired from a prospectively maintained database. Patient records were reviewed for demographic information, splenic injury grades, embolisation efficacy, complications, and associated injuries and mortality. Data relating to Injury Severity Scores (ISS) and postprocedural practice (vaccinations, antibiotic prescribing, follow-up imaging) were also obtained.

**Results:**

Thirty-six patients (24 male, 12 female) with a median age of 42.5 years (range 13–97 years) were identified. American Association for the Surgery of Trauma splenic injury grades were III (*n* = 7), IV (*n* = 20) and V (*n* = 9). Seventeen patients had isolated splenic injury and 19 had additional injuries to other organ systems. Median ISS was 18.5 (range 5–50). SAE succeeded first time in 35/36 cases, and upon the second attempt in 1/36 cases. No patients died because of splenic injury or SAE although four patients with polytrauma died owing to other injuries. SAE complications occurred in 4/36 cases. For survivors, vaccinations were administered in 17/32 cases, and long-term antibiotics were initiated in 14/32 cases. Formal follow-up imaging was arranged in 9/32 cases.

**Conclusions:**

These data show that SAE is an effective means of controlling splenic haemorrhage secondary to blunt trauma with no patient requiring subsequent laparotomy. Major complications occurred in 11% of cases. Follow-up practice varied regarding further imaging, antibiotic and vaccination administration.

## Introduction

The spleen is a highly vascular organ with roles in haematopoiesis and immunological function.^1^ Its vascular nature makes it susceptible to injury and significant haemorrhage. In high-income countries, major trauma is the leading cause of death in those aged 5–44 years.^2^ Splenic injury is an important sequela of abdominal trauma and thus remains a leading cause of morbidity and mortality in this population.^3^

Over the past quarter century, the management of splenic trauma has altered significantly as operative management becomes increasingly infrequent.^4^ Before the turn of the millennium, it was noted that 65% of blunt splenic trauma could be managed non-operatively with a 98% success rate and the avoidance of excess morbidity or mortality.^5^ Non-operative management (NOM) is now considered the gold standard in haemodynamically stable patients with blunt splenic trauma who do not present with peritonitis or other injuries requiring laparotomy.^6^

**Figure 1:**
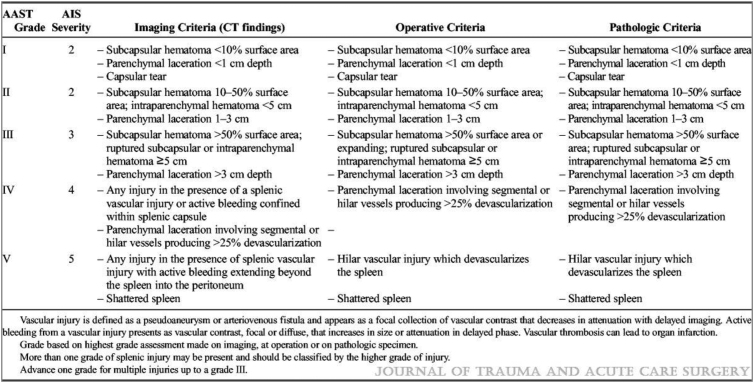
Spleen organ injury scale – 2018 revision AAST = American Association for the Surgery of Trauma; AIS = abbreviated injury scale; CT = computed tomography^9^

This shift in approach has been helped by advances in dual-phase computed tomography (CT) imaging, which has enabled precise identification and grading of splenic injury.^3,7^ A 2006 study revealed that modern CT demonstrates 100% sensitivity and 88% specificity in predicting the need for intervention in splenic trauma.^8^ The American Association for the Surgery of Trauma (AAST) constructed a grading system for splenic trauma in 1987, most recently revised in 2018, which is detailed in Figure 1.^9^

Other radiological advances have facilitated the trend towards NOM. Splenic artery embolisation (SAE) is a minimally invasive, endovascular, interventional radiology (IR) procedure that uses a variety of embolic agents including absorbable gelatin foam, particles (300–500mm), vascular plugs and/or coils to embolise the splenic artery.^10^ First described in 1981, it has burgeoned in popularity as IR facilities become increasingly widespread. In 2012, the Eastern Association for the Surgery of Trauma (EAST) produced guidelines for managing splenic injury and suggested that angiography and SAE should be considered in cases described as AAST grade III and above where contrast blush or moderate haemoperitoneum is present, or where there is evidence of ongoing splenic bleeding.^11^

Despite the rapidly expanding literature regarding SAE and its role in splenic trauma, the EAST guideline authors noted that many questions regarding the NOM of splenic injuries remain unanswered; for example, whether repeated imaging has any value, if SAE leads to an immunological deficiency, and whether such patients require vaccinations and/or long-term prophylactic antibiotics.

The aim of this paper was to investigate outcomes and postprocedural management of patients who underwent SAE for blunt splenic trauma at a UK trauma centre.

## Methods

This was a single-centre study based in a UK hospital that was granted trauma centre status in 2018. Prior to the commencement of data collection Caldicott approval was obtained.

CDN Radiology Information System (CRIS) codes for IR embolisation procedures were used to identify all cases of SAE performed for trauma between January 2012 and January 2022.

Patient records, both physical and electronic, were reviewed for demographic data, details of trauma, grade of splenic injury, success of embolisation, complications and associated injuries and mortality.

Data relating to vaccination administration, prophylactic antibiotic prescribing, and the use of follow-up imaging were also obtained from patient records. Injury Severity Scores (ISS) were obtained from the Scottish Trauma Audit Group. Study data were collated using a Microsoft Excel database.

## Results

Thirty-six patients were identified during the study period, 24 male and 12 female. Patients’ ages ranged from 13 to 97 years with a median of 42.5 years. The various mechanisms of injury are detailed in Table 1. The AAST splenic injury grades involved were III (*n* = 7), IV (*n* = 20) and V (*n* = 9). Seventeen of the 36 patients had isolated splenic injury and the remainder had additional injuries to other organ systems.

**Table 1:** Mechanisms of blunt splenic injury

Mechanism	Cases, *n*
Vehicle–vehicle road traffic accident	11 (30%)
Vehicle–pedestrian road traffic accident	2 (5%)
Fall	16 (43%)
Bicycle accident	7 (19%)

Four patients did not survive to discharge. These patients had all sustained multiple injuries and had a median ISS of 36.5 (range 16–50). Non-survivors had a median age of 75 years (range 41–97) and a median age-adjusted Charlson Comorbidity Index of 7 (range 0–7). The median ISS value for survivors was 17.5 (range 5–43). Survivors had a median age of 40.5 years (range 13–89 years) and a median age-adjusted Charlson Comorbidity Index of 0 (range 0–6).

In terms of the non-survivors, one patient died of a stroke following operative orthopaedic intervention for multiple limb injuries. A further patient suffered catastrophic diffuse axonal injury, leading to the decision to withdraw life support on the intensive care unit. Another patient died from multiorgan failure secondary to decompensated liver disease. The final mortality was due to hospital-acquired pneumonia secondary to chest wall trauma.

SAE avoided further invasive intervention for management of splenic trauma in 35/36 cases. In the remaining case, CT imaging was performed 2 days post SAE because of increasing abdominal pain. It revealed some filling of splenic artery branches distal to the coil and active extravasation. A second attempt at SAE was successfully performed utilising absorbable gelatin.

Major complications secondary to SAE arose in 4/36 cases. The most common complication (*n* = 3) was splenic abscess. Splenic abscesses were associated with considerable morbidity, requiring CT-guided drainage over multiple admissions. The other major complication (*n* = 1) was an iatrogenic external iliac artery pseudoaneurysm secondary to percutaneous transcatheter insertion. This required a further endovascular procedure to stent the pseudoaneurysm.

Three patients were anticoagulated at the time of presentation. In all three cases the relevant anticoagulant medication was immediately withheld. One patient on warfarin for atrial fibrillation had their anticoagulation promptly reversed. They later died in the intensive care unit secondary to diffuse axonal injury. A 97-year-old patient had their apixaban permanently discontinued as the risk-to-benefit ratio was deemed unfavourable. Another patient on apixaban for a previous deep vein thrombosis had this medicine withheld during their admission and recommenced at discharge.

For survivors, vaccinations were administered in 17/32 cases and prophylactic long-term antibiotics were prescribed in 14/32 cases. Two patients stopped taking long-term antibiotics because of side-effects. A further two patients had their antibiotics stopped following review of their outpatient follow-up imaging.

Thirty cases were available for long-term follow-up for infective complications related to reduced splenic function. This excluded the four cases who did not survive to discharge, as well as two cases that involved out-of-region patients. In all 30 cases, no evidence of readmission due to systemic infective complications was noted. Follow-up periods ranged from 4 to 489 weeks with a median of 198 weeks.

Formal outpatient follow-up imaging was arranged in 9/32 cases. Five patients underwent further CT imaging; four patients had abdominal ultrasound. One patient had formal follow-up CT as an inpatient. The remaining eight patients had outpatient imaging at a median of 44 (range 21–365) days post SAE. In one of the outpatient CT scans a complication was identified (splenic abscess) but all other follow-up imaging was normal. In all nine cases, viable splenic tissue was identified by a consultant radiologist.

## Discussion

These data show that SAE is an effective method of controlling bleeding in patients presenting with AAST grade III to V splenic injuries. All patients avoided laparotomy and the associated morbidity and mortality. Although a mortality rate of 11% was noted, no fatalities were a direct consequence of splenic haemorrhage.

By comparison, an Australian retrospective paper showed open splenectomy for an isolated splenic injury to have a 10% mortality rate and a 25% complication rate.^12^ A British study also revealed considerable mortality (13.5%) and morbidity (21.6%) following emergency splenectomy.^13^ It has previously been demonstrated that those patients suitable for NOM have much better outcomes.^14^

This study also demonstrated that SAE is associated with a moderate risk of major complications (11%). However, this is in keeping with the audit standard of the Society of Interventional Radiology who suggest complication rates should be less than 15%.^15^

Anticoagulated patients were managed uniformly with all three having their anticoagulant withheld following the diagnosis of splenic injury. The solitary patient receiving warfarin had their anticoagulation reversed on admission, as recommended by British Committee for Standards in Haematology (BCSH) guidance.^16^ The two patients on apixaban did not receive reversal with andexanet alfa. National Institute for Health and Care Excellence guidance states that there is insufficient evidence to recommend this with the exception of major bleeding originating from the gastrointestinal tract.^17^

Great variability was noted in terms of follow-up imaging, which was organised for 28% of surviving patients. This finding reflects the uncertainty in the literature regarding this issue. In 2005, a survey of EAST members found that the vast majority (85.5%) did not advocate follow-up radiology.^18^ The 2012 EAST guidelines noted “necessity of repeated imaging” as a particular topic for future study.^11^ To this day, there remains no absolute evidence-based standard that can guide practice in this area. A Delphi study from 2013, involving 30 expert trauma surgeons and interventional radiologists worldwide, came to the consensus (88%) that routine post-discharge imaging is not indicated.^19^

Comparable uncertainty was also found regarding the use of prophylactic antibiotics and vaccinations in those who survived to discharge. Long-term prophylactic antibiotics were prescribed for 44% of cases (*n* = 14) and vaccinations were administered in 53% of cases (*n* = 17). The split practice observed in this study is not a unique phenomenon. A US study in which Michigan trauma surgeons were surveyed with regards to their practices post SAE revealed that 51% vaccinated such patients and 49% did not.^20^ Such inconsistencies in practice reflect the lack of best practice guidance. Again the 2012 EAST guidelines question the need for vaccination post embolisation and the immunological consequences of SAE as areas for future study.^11^

The value of vaccination and prophylactic antibiotics utilisation post embolisation depends upon the degree of splenic immunological function that is preserved. It is well established that post splenectomy, patients are at risk of encapsulated organisms such as *Streptococcus pneumoniae* which can lead to overwhelming post-splenectomy infection (OPSI) and fatal sepsis.^21^ In light of this, 2011 guidance from the BCSH states that vaccinations should be provided to “all splenectomised patients and those with functional hyposplenism”, which they state includes “therapeutic splenic embolisation”.^22^ Within this patient group, BCSH also advocate for lifelong prophylactic antibiotic administration in high-risk groups such as those aged <16 or >50, those with a history of inadequate serological response to vaccination or invasive pneumococcal disease.^22^ For the remainder of the patient population they advise a “supply of appropriate antibiotics for emergency use”.^22^

Nonetheless, despite this national guidance, there is now growing evidence that splenic immunological function may well remain intact post embolisation.^23^ A 2016 systematic review examined 12 studies and found that all but one suggested preserved splenic function post SAE. In addition, not a single episode of OPSI was recorded within these studies.^24^ In 2015 Foley *et al* noted that immunoglobulin M (IgM) memory B cell levels, a marker for splenic function, were markedly increased at 6 months in those patients who had SAE rather than splenectomy.^25^ A subsequent follow-up of this cohort found that at a median of 8.5 years post embolisation, these patients had normal levels of IgM memory B cells.^26^ Although the present study involves limited numbers, it is interesting to note that not a single patient was readmitted with infective complications potentially related to a reduction in splenic function.

Despite these recent developments that may suggest that postprocedural vaccinations are unnecessary there is a still a lack of high-quality, reproducible evidence to support this policy.^23^ Clearly there is a requirement for further research in this area to guide best practice and enable health institutions to derive evidence-based protocols for patients who undergo SAE.

To help address these issues it would be useful to identify a biomarker that can reliably indicate active splenic function. Schimmer *et al* note Howell–Jolly bodies, a cytopathological marker of impaired splenic function, lack sensitivity in detecting mild hyposplenism.^24^ The red blood cell (RBC) pit test, as described by Falimirski *et al*, is an alternate option.^27^ In their study of 40 splenic trauma patients with grade IV or V injuries, a statistically significant difference (*p* = 0.0002) was found between average RBC pit levels in those whose injuries were managed conservatively vs those that had splenectomy. However, they did not specify whether any patient in the non-operative group underwent SAE.

It is unfortunate that little progress has been made in determining the most appropriate postprocedural management of SAE patients. Avoiding vaccination and long-term antibiotics can help minimise healthcare costs and promote appropriate antimicrobial stewardship. It has been postulated that by the year 2050, 10 million people may die annually because of antibiotic resistance.^28^ Hence reducing unnecessary usage wherever possible is crucial in trying to address this problem. In addition, minimising the use of antibiotics decreases the potential for patients to suffer iatrogenic harm as a result of side-effects and drug interactions.

## Conclusions

In summary this study shows that SAE is an effective means of controlling splenic haemorrhage, with no patient requiring subsequent laparotomy. Major complications were not infrequent and occurred in 11% of cases. Great variance was found in terms of follow-up practice regarding further imaging, antibiotics and vaccinations. This is unsurprising owing to the lack of good evidence in these areas. A recent systematic review has suggested that one could anticipate maintained splenic function post SAE.^24^ Nevertheless, until high-quality evidence is generated, clinical practice in these areas will continue to be inconsistent. Having a greater understanding of splenic function post SAE offers the opportunity to decrease healthcare costs and improve antimicrobial stewardship.
